# Regular Irregulars

**Published:** 1851-09

**Authors:** 


*Regular Irregulars.*—The phrase forming the caption of this communication, occurred in an extract from the Lancet copied into a former number of the Stethoscope. It is a very expressive title of a class of medical practitioners, such as Hannah More describes in the Christian Church under the term Borderers. They keep themselves within the ranks of the regulars, but at the same time border as closely as possible on those of the irregulars. They are very careful to do nothing which would subject them to expulsion or even to discipline, and talk very emphatically about the importance of a rigid adherence to the code of ethics. Their uniform is made of the most showy, glittering materials. If, however, their *manœuvres* be watched, they will be seen edging along very closely to the irregulars and picking off a button or so, which, when fastened on their own regimentals is concealed from common observation by the general glare. At the same time that they are very loud in their denunciations of the artifices of the irregulars, they do not scruple to steal a little trick, now and then, which they may use to advantage.

Dr. A. hates a Thompsonian, and despises a Homœopath. He is very strict in his outward conduct towards a brother practitioner. If the latter, however, be a young man, he treats him with an air so compounded of respect and patronage, that the patient and friends give him great credit for his *tenderness towards the ignorant and inexperienced youth!* Perhaps the latter may be green enough to receive it in the same manner, and feel quite flattered by the condescension of one so vastly his superior in skill and statio. The distinguished *savan* informs the patient and friends that the physician has treated the case with a remarkable degree of skill — indeed quite as much so *as if the patient had been under his own care!* Of course they all feel quite relieved by this assurance, but agree that it would be safer to trust this superior skill and talent on the next occasion.

Dr. A. has a patient who is rather unwell, and he prescribes a little ride and some cheerful company. A visit to a friend would be very renovating to the nervous system. A seat is offered in Dr. A.'s carriage, and Mrs. B. is quite captivated by the Dr.'s urbanity and obliging disposition. How much better to employ Dr. A. than Dr. C., who don't keep a carriage, and simply prescribes for his patients without adding all these little valuable courtesies. Mrs. B. rides by the Dr.'s side to Mrs. D.'s, and there all the family come out to receive her. The Dr. would not go in for the world, lest he should be considered as endeavoring to supplant the family doctor. However, he is duly introduced by Mrs. B. amidst many flattering remarks, and he rides off, congratulating himself on his *scrupulous observance of professional etiquette.* Mrs. B. finds one of Mrs. D.'s children an invalid, who has been a long time under medical treatment, and all agree that it would be best to *try Dr. A.'s skill*, and turn off the old faithful family physician.

Mrs. E. has been ailing a good while. Her appetite is very bad. She cannot relish anything. Dr. A. calls at ———'s restorative and orders some nice little nick-nacks to be sent to Mrs. E. "What a capital doctor," exclaims Mrs. E., "so considerate and liberal;" and she lauds him to the skies among her acquaintance, with a voice improved in clearness and strength by broiled partridge and fixings, &c.

Mrs. F. has disordered bowels — she really cannot keep anything upon her stomach. While in this deplorable condition and disgusted with life itself, a finely-dressed servant enters, bearing a tray covered with a white napkin, and presents it, with Dr. A.'s compliments. "What other doctor," exclaims Mrs. F., "would have been so attentive?" Just at that moment Mrs. G. drops in to inquire after Mrs. F.'s health. The feminine curiosity of both is excited to ascertain the nature of the nicety sent by the generous and considerate doctor. The napkin is raised, and an elegant glass is disclosed, packed in crushed ice, and filled with some beverage sparkling as if fresh from a fountain. A taste proves it to be iced champaigne. "What an elegant doctor!" they both exclaim. Besides this impression, there is another very profitable one. The popular proprietor of the restorative and all his menials, are made acquainted with the important fact that Mrs. E. and Mrs. F., &c., employ Dr. A.; and besides this, as one good turn deserves another, mine host becomes an advertiser of Dr. A. to all his customers.

Mrs. H. employs Dr. A. to attend herself, and is quite captivated by a round of these pretty little civilities. Her husband requires medical attention and sends for Dr. J., and is very well satisfied with the treatment of his case. Dr. A. is informed of the fact at his next visit. Alarmed and envious at the effect of such competition, he exclaims, "Why did you not send for me?" The next time Mr. H. is taken sick, like a dutiful spouse, he permits Mrs. H. to send for her favorite doctor.

Now what would be the result if the whole medical profession were to adopt this mode of purchasing practice? He who should degrade himself the most by playing the sycophant to his patients, and make them the most costly presents, would render himself the most popular physician. The patient would learn to set a low estimate upon professional skill, and look upon all these expensive courtesies as perquisites, instead of regarding the services of the physician as of far more value than the fee which is expected in return. These are a few small scraps of the worn-out regimentals of the irregulars with which Dr. A. patches his own. Unfortunately for himself, however, he gradually puts on so many, that at last every shred of his own flashy livery is replaced by that of his antagonists, like the silk stocking which was darned with cotton so often, that it finally became a cotton stocking. He then shines out in his true livery, and is expelled from the society of those who had long suspected and now detest him.

If any one shall perceive from this sketch that he is unconsciously pursuing such a course towards his own ruin, let him pause and resolutely determine no longer to hang about the out-skirts of the regular phalanx, but take a bold, manly stand in favor of high, independent principles.—*Stethoscope.*

